# Femoral Head Reduction Osteotomy for Legg-Calvé-Perthes Disease Sequelae: Case Report

**DOI:** 10.1055/s-0042-1758365

**Published:** 2023-07-31

**Authors:** Tiago Fontainhas, David Pereira, Ana Sofia Costa, Rui Sousa, Ana Flávia Resende, Joaquim Nelas

**Affiliations:** 1 Departamento de Ortopedia e Traumatologia, Centro Hospitalar Tondela-Viseu, Viseu, Portugal

**Keywords:** femur head, Legg-Calvé-Perthes disease, osteotomy

## Abstract

Legg-Calvé-Perthes disease (LCPD) commonly causes sequelae in the hip joint morphology. A common variant is an oversized, nonspherical femoral head, associated with a short femoral neck and elevated greater trochanter, which leads to femoroacetabular impingement (FAI). The innovative Ganz technique for surgical hip dislocation opened up new treatment possibilities for FAI, including LCPD sequelae, without increasing the risk of avascular necrosis of the femoral head. In the ellipsoid coxa magna resulting from LCPD, joint wear is more accentuated in the central portion of the femoral head; the lateral third remains intact as it does not articulate with the acetabulum. A femoral head reduction osteotomy technique developed for such cases resects the damaged portion of the femoral head and restores its sphericity. Short-term outcomes are encouraging. The present case report presents a patient with LCPD sequelae submitted to a femoral head reduction osteotomy.

## Introduction

Legg-Calvé-Perthes Disease (LCPD) commonly causes sequelae in the hip joint morphology. 1 A common post-LCPD variant is an oversized nonspherical femoral head (coxa magna), associated with a short femoral neck (coxa brevis) and an elevated greater trochanter (GT), leading to femoroacetabular impingement (FAI). 1 2 Surgical procedures to correct FAI deformities and the premature wear of the hip joint may avoid long-term sequelae. 1 3 The introduction of the safe hip dislocation technique by Ganz et al. 4 revolutionized FAI treatment as it allowed full hip exposure, dynamic visualization of the affected area, and targeted treatment without increasing the risk of avascular necrosis (AVN) of the femoral head. 5 In the LCPD typical ellipsoid coxa magna, joint wear is more accentuated in the central portion of the head, and the lateral third is intact, as it does not articulate with the acetabulum. 1 For these cases, Ganz et al. 6 7 developed the femoral head reduction osteotomy (FHRO) technique, which takes advantage of the safe hip dislocation approach. This technique creates a subperiosteal flap to protect the retinaculum, resects the damaged central portion of the femoral head, and restores its sphericity. 6 7 8 This report presents the case of an adolescent with LCPD sequelae treated with FHRO and highlights this still relatively unknown surgical technique.

## Case Report

**Fig. 1:**
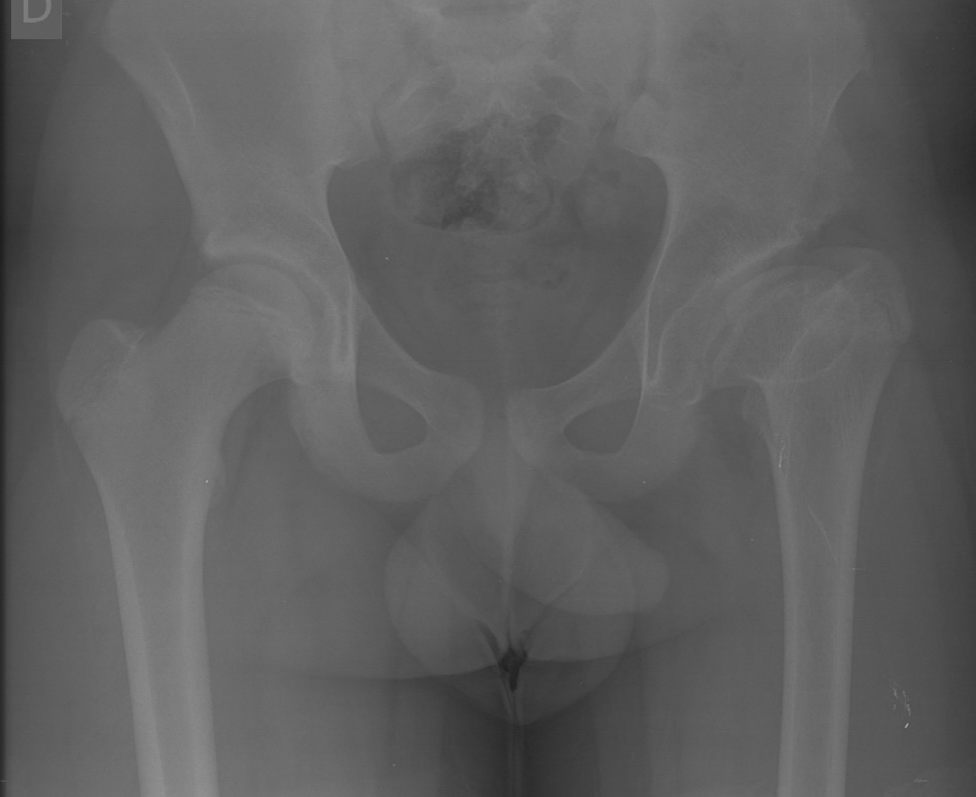
Preoperative radiograph showing morphological sequelae of Legg-Calvé-Perthes disease in the left hip.

A 15-year-old boy was referred to a Pediatric Orthopedics visit due to left coxalgia and claudication for 6 months. The patient had no history of trauma or fever and showed no improvement with medication or activity restriction. He had a personal history of LCPD in the left hip and underwent conservative treatment. On physical examination, he had pain during flexion and internal rotation and complete mobility in flexion and extension but decreased mobility in internal and external rotation. The patient also presented claudication and a Trendelenburg gait. Radiographs revealed a congruent left hip with morphological changes as LCPD sequelae, namely an oversized ellipsoid femoral head, GT elevation, shortened femoral neck, and mild acetabular dysplasia. ( Fig. 1 ).

**Fig. 2:**
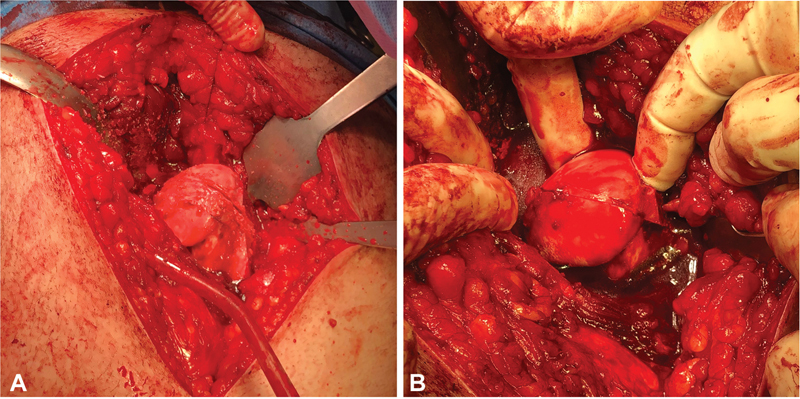
Intraoperative photography. **(A)** Ellipsoid femoral head with wear in the central portion. The sections referred to the reduction osteotomy of the femoral head. **(B)** We reduced the femoral head by removing the central bone block, leaving it with a more spherical shape.

**Fig. 3:**
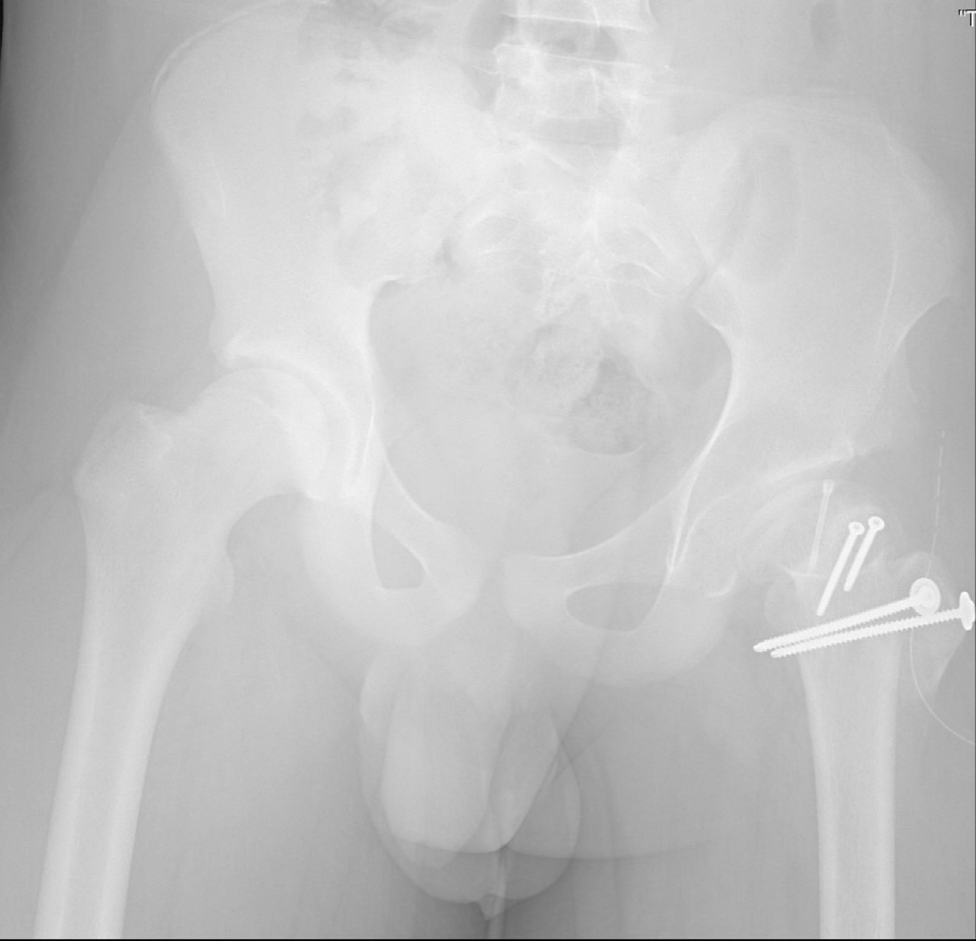
Immediate postoperative radiograph. Preoperative Southwick angle = Ângulo pré-operatório de SouthwickAbsent = AusentesPresent = PresentesComplications (12 months) = Complicações (12 meses)

The patient underwent FHRO and GT distalization with no intercurrences. We performed the Ganz technique of safe dislocation by GT osteotomy. The ellipsoid morphology of the femoral head, chondral lesions in the central portion, and cartilage integrity in the medial and lateral thirds were all confirmed. We mobilized a subperiosteal retinaculum flap to protect the blood supply to the femoral head. Then, we proceeded to the head reduction osteotomy using two vertical sections to divide it into three portions ( Fig. 2a ). We resected the central third and reduced the femoral head ( Fig. 2b ), fixating it with two cortical screws to obtain the final spherical shape. Intraoperative bleeding from the femoral head confirmed the integrity of its vascularization. We inspected the acetabulum and found no osteochondral or labrum lesions. Finally, the GT was mobilized distally and fixed with two cortical screws ( Fig. 3 ).

**Fig. 4:**
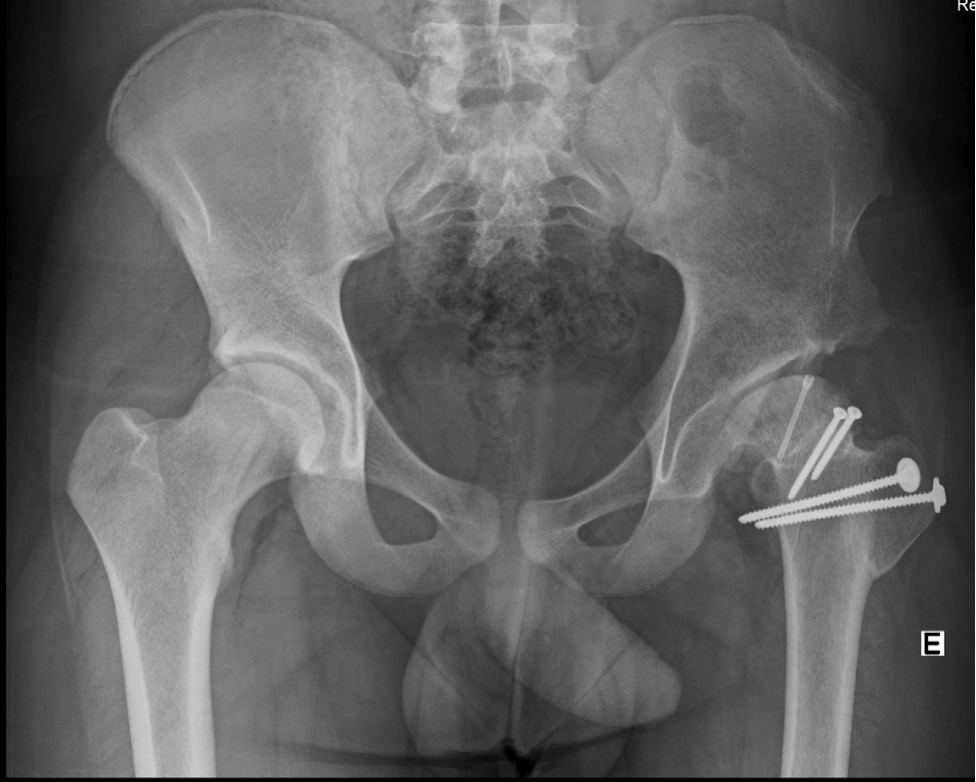
Follow-up at 12 months showing bone consolidation and no signs of avascular necrosis of the femoral head.

The postoperative period was uneventful. The patient started walking with crutches with no load at 48 hours. We did not allow adduction beyond midline or active external rotation before 6 weeks. Partial loading started at 12 weeks after radiographic confirmation of bone healing. At 12 months, the patient had no complaints or limitations, and there was bone consolidation with no AVN of the femoral head ( Fig. 4 ).

## Discussion

Legg-Calvé-Perthes Disease commonly causes morphological sequelae of the hip joint. A frequent variant is the oversized nonspherical femoral head, resulting in FAI due to a hinge effect between the lateral wall of the acetabulum and the central portion of the head. In these cases, the medial and lateral thirds are often intact. 1 7 Based on this observation, Ganz et al. 6 7 developed the FHRO technique, which resects the degenerated central third of the femoral head, restores its sphericity, and resolves the FAI, reducing the risk of AVN. Since its introduction, three series showed encouraging short-term results. In 2010, Ganz et al. 7 published a series of 14 patients undergoing FHRO since 2001, 13 due to LCPD sequelae. There was no report of AVN in this series. Although outcomes were satisfactory, only one case did not require adjuvant periacetabular osteotomy (PAO) due to insufficient acetabular coverage or instability. 7 In the largest series in the literature, Paley 9 reported 21 hips submitted to FHRO. All patients had coxalgia, claudication, and a positive Trendelenburg sign. One hip was excluded from the series due to an iatrogenic fracture of the femoral neck, resulting in a conversion to total hip arthroplasty. The average diameter of the femoral head decreased from 133 to 96% compared with the normal contralateral hip. Five patients required a pelvic osteotomy. Another five patients required an articulated external fixator because of femoral head subluxation. A single case had AVN at 18 weeks of follow-up. All patients with good flexion and extension preoperatively had good to excellent mobility after the procedure. Eight patients had significant preoperative stiffness and did not improve significantly after 1 year of follow-up. However, five out of these eight patients reported complete pain resolution. Based on his study, Paley defines that the best indications for FHRO include a nonspherical femoral head, good previous mobility in flexion and extension, and limited cartilaginous lesion in the acetabulum with corresponding degeneration on the resectable femoral side. According to Paley, several factors may have contributed to the single AVN observed in the study. This case was the largest resection from the series (37% of the femoral head) and the only patient with open physis at the time of FHRO and a previous femoral osteotomy.

Seibenrock et al. 10 published a series of 11 hips submitted to FHRO with a minimum follow-up period of 3 years. Five required another intervention to improve joint congruence after an average time of 2.3 years. Besides heterotopic calcification, there were no complications.

In summary, FHRO is an evolving and technically challenging procedure, even in experienced hands, but with encouraging short-term outcomes for patients with nonspherical femoral heads as LCPD sequelae. However, further studies are required to define indications, contraindications, and long-term results of FHRO.
