# Supplementary material for: Femoral Head Reduction Osteotomy for Legg-Calvé-Perthes Disease Sequelae: Case Report
Source: Rev Bras Ortop (Sao Paulo). 2023 Jul 31;59(Suppl 1):e94–7. doi: 10.1055/s-0042-1758365 (PMC11254446; doi:10.1055/s-0042-1758365)
Supplement: Related texts

# Research article: Osteotomia de redução da cabeça femoral por sequelas de doença de Legg-Calvé-Perthes: Relato de caso

**Keywords:** cabeça do fêmur, doença de Legg-Calvé-Perthes, osteotomia

## Abstract

A doença de Legg-Calvé-Perthes (DLCP) origina comumente sequelas na morfologia da articulação coxofemoral. Uma variante comum é a cabeça do fêmur não-esférica e sobredimensionada, associada a um colo femoral curto e grande trocânter elevado, que origina conflito femuroacetabular (CFA). A inovadora técnica de luxação cirúrgica do quadril de Ganz abriu novas possibilidades de tratamento para CFA, incluindo a originada por sequelas de DLCP, sem aumentar o risco de necrose avascular da cabeça femoral. Na coxa magna elipsoide resultante de DLCP, constatou-se que o desgaste articular era mais acentuado na porção central da cabeça do fêmur e que o terço lateral está íntegro, pois não articula com o acetábulo. Para estes casos, foi desenvolvida a técnica de osteotomia de redução da cabeça femoral, que resseca a porção danificada da cabeça femoral e restabelece a sua esfericidade. Os resultados a curto prazo são encorajadores. O presente relato de caso apresenta um paciente com sequelas de DLCP tratado com osteotomia de redução da cabeça femoral.

## Introdução

A doença de Legg-Calvé-Perthes (DLCP) comumente origina sequelas na morfologia da articulação coxofemoral. 1 Uma variante comum após DLCP é a cabeça do fêmur não-esférica sobredimensionada (coxa magna), associada a colo femoral curto (coxa brevis) e grande trocânter (GT) elevado, o que origina conflito femuroacetabular (CFA). 1 2 Para evitar sequelas a longo-prazo, procedimentos cirúrgicos foram desenvolvidos para corrigir as deformidades responsáveis pelo CFA e o desgaste prematuro da articulação do quadril. 1 3 A introdução da técnica de luxação segura do quadril por Ganz et al. 4 revolucionou o tratamento de CFA, pois permitiu a exposição total do quadril, visualização dinâmica da área de conflito e tratamento dirigido, sem aumentar o risco de necrose avascular (NAV) da cabeça do fêmur. 5 Na coxa magna elipsoide típica da DLCP verificou-se que o desgaste articular era mais acentuado na porção central da cabeça e que o terço lateral está íntegro, pois não articula com o acetábulo. 1 Para estes casos, Ganz et al. 6 7 desenvolveram a técnica de osteotomia de redução da cabeça femoral (ORCF), que aproveita a abordagem da luxação segura do quadril, cria um retalho subperiosteal para proteção do retináculo, resseca a porção central danificada da cabeça femoral e restabelece a sua esfericidade. 6 7 8 O presente relato de caso apresenta um adolescente com sequelas de DLCP tratado com ORCF e destaca esta técnica cirúrgica ainda relativamente pouco divulgada.

## Relato de Caso

Rapaz de 15 anos, referenciado à consulta de Ortopedia Pediátrica por coxalgia esquerda e claudicação da marcha com 6 meses de evolução, sem história de traumatismo ou febre, sem melhoria com medicação ou restrição de atividades. Tinha antecedentes pessoais de DLCP no quadril esquerdo, tratada conservadoramente. No exame físico, apresentava dor com flexão e rotação interna, mobilidade completa em flexão-extensão, mas diminuída em rotação interna e externa. Apresentava claudicação e marcha de Trendelemburg. Radiografias revelam um quadril esquerdo congruente com alterações morfológicas por sequelas de DLCP, nomeadamente uma cabeça femoral sobredimensionada elipsoide, elevação do GT, colo do fêmur encurtado e displasia acetabular ligeira ( Fig. 1 ).

**Fig. 1:** Radiografia prȁ-operatória, demonstrando sequelas morfológicas de doença Legg-Calvé-Perthes no quadril esquerdo.

O doente foi submetido a ORCF e distalização do GT, sem intercorrências. A técnica de luxação segura do quadril de Ganz, por osteotomia do GT, foi realizada. Confirmou-se a morfologia elipsoide da cabeça femoral, a presença de lesões condrais da porção central e a integridade da cartilagem nos terços medial e lateral. Desenvolveu-se o retalho subperiosteal do retináculo, para mobilização e proteção do suprimento sanguíneo da cabeça femoral. Seguidamente, procedeu-se à osteotomia de redução da cabeça, através de dois cortes verticais dividindo-a em três porções ( Fig. 2a ). O terço central foi ressecado e a cabeça do fêmur foi reduzida ( Fig. 2b ) e fixada com dois parafusos corticais, obtendo-se um formato esférico final. Sangramento intraoperatório da cabeça confirmou a integridade da sua vascularização. O acetábulo foi inspecionado, não se detectando lesões osteocondrais ou do labrum. Por fim, o GT foi mobilizado distalmente e fixado com dois parafusos corticais ( Fig. 3 ).

**Fig. 2:** Fotografia intraoperatória. ( **A)** Cabeça elipsoide com desgaste na porção central. Realizados cortes para osteotomia de redução da cabeça femoral. ( **B)** Após remoção do bloco ósseo central, a cabeça é reduzida e apresenta agora um formato mais esférico.

**Fig. 3:** Radiografia pós-operatória imediata.

O pós-operatório decorreu sem intercorrências. O paciente iniciou marcha com canadianas em descarga às 48 horas. Adução além da linha média e rotação externa ativa não foram permitidas antes das 6 semanas. Carga parcial iniciou-se às 12 semanas após confirmação radiográfica de consolidação óssea. Aos 12 meses, o doente estava sem queixas e sem limitações, e verificou-se consolidação óssea sem NAV da cabeça do fêmur ( Fig. 4 ).

**Fig. 4:** Controle aos 12 meses. Verifica-se consolidação óssea e ausência de sinais de necrose avascular da cabeça femoral.

## Discussão

A DLCP comumente origina sequelas morfológicas da articulação coxofemoral. Uma variante frequente é a cabeça femoral não-esférica sobredimensionada, causando CFA por efeito charneira, entre a parede lateral do acetábulo e a porção central da cabeça. Nestes casos, os terços medial e lateral habitualmente estão íntegros. 1 7 Com base nisso, Ganz et al. 6 7 desenvolveram a técnica de ORCF, que resseca o terço central degenerado da cabeça femoral, restaura a sua esfericidade e resolve o CFA, mantendo o risco baixo de NAV. Desde a sua introdução, três séries foram publicadas na literatura, com resultados encorajadores a curto prazo. Ganz et al. 7 publicaram em 2010 uma casuística de 14 pacientes submetidos a FHRO desde 2001, 13 por sequelas de DLCP. Necrose avascular não foi reportada nesta série. Os resultados foram considerados satisfatórios; contudo, apenas um caso não necessitou de osteotomia periacetabular (OPA) adjuvante, devido a cobertura acetabular insuficiente ou instabilidade. 7 Paley 9 apresentou uma série de 21 quadris submetidos a ORCF, a maior descrita na literatura. Todos os doentes manifestavam coxalgia, claudicação e sinal Trendelemburg positivo. Um quadril foi excluído da casuística por fratura iatrogênica do colo do fêmur, resultando em conversão para artroplastia total do quadril. O diâmetro da cabeça femoral diminuiu de 133 para 96%, em média, em relação ao quadril contralateral normal. Cinco pacientes necessitaram de osteotomia pélvica. Cinco outros pacientes precisaram de fixador externo articulado por subluxação da cabeça femoral. Um caso apresentou NAV às 18 semanas de seguimento, a única da série. Todos os doentes com boa mobilidade prévia em flexo-extensão apresentaram mobilidade boa a excelente no pós-operatório. Oito pacientes tinham rigidez significativa pré-operatoriamente e não melhoraram significativamente após 1 ano de seguimento. Contudo, resolução completa da dor ocorreu em 5 destes 8 pacientes. Baseado no seu estudo, Paley define que as melhores indicações para ORCF são cabeça femoral não-esférica, boa mobilidade prévia em flexão-extensão e lesão cartilaginosa limitada no acetábulo com degeneração correspondente no lado femoral a ressecar. Segundo Paley, vários fatores podem ter contribuído para a única NAV do estudo. Foi a maior resseção da série (37% da cabeça femoral), o único paciente com fises abertas durante a ORCF e o único com osteotomia do fêmur prévia.

Seibenrock et al. 10 publicaram uma casuística de 11 quadris submetidos a ORCF, com seguimento mínimo de 3 anos. Cinco necessitaram de outra intervenção para melhorar a congruência articular após 2,3 anos, em média. Além de uma calcificação heterotópica, não se verificaram complicações.

Em suma, a ORCF é um procedimento em evolução e tecnicamente desafiante mesmo em mãos experientes, mas com resultados encorajadores a curto prazo para pacientes com cabeças do fêmur não-esféricas por sequelas de DLCP. Contudo, mais estudos são necessários para definir as indicações e contraindicações para a sua realização e determinar os resultados a longo prazo.
